# Nonlinear associations of the neutrophil-to-lymphocyte ratio and derived neutrophil-to-lymphocyte ratio with asthma–COPD overlap prevalence: a cross-sectional study

**DOI:** 10.3389/fmed.2026.1950041

**Published:** 2026-09-29

**Authors:** Ruyue Jin, Shuni Wang, Jiajun Zhang, Yonghu Liu, Shaofeng Sun, Jiarong Zhu, Xuexia Tong, Peng Zhang

**Affiliations:** Department of Pulmonary and Critical Care Medicine, General Hospital of Ningxia Medical University, Yinchuan, China

**Keywords:** asthma–COPD overlap, derived neutrophil-to-lymphocyte ratio, neutrophil-to-lymphocyte ratio, restricted cubic spline, systemic inflammation, threshold effect

## Abstract

**Background:**

Asthma–chronic obstructive pulmonary disease overlap (ACO) is associated with persistent airflow limitation and adverse clinical outcomes. Whether the neutrophil-to-lymphocyte ratio (NLR) and derived NLR (dNLR) show nonlinear associations with ACO prevalence among patients with asthma is uncertain.

**Methods:**

We conducted a retrospective cross-sectional analysis of 505 patients with asthma. ACO was defined by coexisting physician-documented diagnoses of asthma and COPD. The usual diagnostic framework is based on Global Initiative for Asthma guidance for asthma and Global Initiative for Chronic Obstructive Lung Disease criteria for COPD; the latter include compatible clinical features and persistent post-bronchodilator airflow limitation (FEV1/FVC <0.70). NLR and dNLR were evaluated as continuous, standardized, and quartile-based exposures. Multivariable logistic regression, restricted cubic splines, and two-piecewise logistic regression were used to assess associations and potential inflection points. Medication-stratified analyses were exploratory.

**Results:**

Of 680 patients screened, 505 were included, including 190 (37.6%) with ACO and 315 (62.4%) with asthma without COPD. In the fully adjusted model, the highest vs. lowest quartiles of NLR [odds ratio [OR], 4.97; 95% confidence interval [CI], 2.50–9.87; *P* < 0.001] and dNLR (OR, 3.31; 95% CI, 1.71–6.41; *P* < 0.001) were associated with higher ACO prevalence. Nonlinearity was detected for both markers (*P* for nonlinearity = 0.023 for each). Piecewise models identified inflection points at NLR 2.0 and dNLR 1.3; the associations were steeper below these values and remained positive but attenuated above them. Exploratory interaction tests were significant for inhaled corticosteroid, long-acting muscarinic antagonist, and inhaled corticosteroid plus long-acting *β*2-agonist use.

**Conclusion:**

Higher NLR and dNLR were independently associated with greater ACO prevalence in this single-center asthma cohort. These inexpensive blood-derived indices may help identify patients who warrant further diagnostic assessment, but the proposed inflection points and medication interactions require external and prospective validation.

## Introduction

Asthma and chronic obstructive pulmonary disease (COPD) are common chronic airway diseases with different but overlapping clinical and inflammatory features (1, 2). Some patients with asthma also have persistent COPD features and are described as having asthma–COPD overlap (ACO) (3). Estimates of ACO prevalence vary substantially because populations and definitions differ (4). Compared with asthma or COPD alone, ACO has been associated with more frequent exacerbations, greater health care use, and poorer health-related quality of life (5). Accessible biomarkers that help identify patients who may require additional evaluation could therefore be clinically useful.

The neutrophil-to-lymphocyte ratio (NLR) and derived NLR (dNLR) are inexpensive indices calculated from a complete blood count and are used as markers of systemic inflammation (6, 7). NLR combines the absolute neutrophil and lymphocyte counts, whereas dNLR is calculated as the neutrophil count divided by the difference between the total white blood cell and neutrophil counts (8, 9). Higher NLR has been associated with disease severity or adverse outcomes in COPD and other conditions (10, 11), and a recent meta-analysis evaluated NLR in asthma (12). Previous studies have also examined blood-cell-derived inflammatory indices in ACO and related respiratory disorders (13–15). However, a linear model may not adequately describe the association across the full biomarker range.

We therefore evaluated the associations of NLR and dNLR with ACO prevalence in a hospital-based cohort of patients with asthma. We used restricted cubic spline and piecewise logistic regression models to assess nonlinearity and estimate data-derived inflection points. We also conducted exploratory analyses to determine whether the associations differed according to recorded maintenance medication use.

## Materials and methods

### Study design and population

We conducted a retrospective cross-sectional study of patients with a documented primary diagnosis of asthma who were evaluated between January 2023 and January 2026 at the General Hospital of Ningxia Medical University. Cough-variant asthma vs. other asthma was a secondary record-based subtype and did not define study eligibility. Inflammatory profiling was limited to a peripheral-blood eosinophil classification; induced-sputum cytology and fractional exhaled nitric oxide (FeNO) were not available. Age at asthma onset was estimated from age and disease duration. Allergy history was considered as a comorbidity-related characteristic because it represents a spectrum of coexisting allergic diseases that are common in patients with asthma. The study was approved by the institutional review board of the General Hospital of Ningxia Medical University (KYLL−2025-1724), which waived the requirement for written informed consent. Asthma and COPD diagnoses were abstracted from clinical records. Asthma was diagnosed in accordance with Global Initiative for Asthma guidance. COPD was identified from the treating physician's diagnosis; under Global Initiative for Chronic Obstructive Lung Disease recommendations, the usual diagnostic standard comprises compatible respiratory symptoms and/or relevant exposure history together with persistent post-bronchodilator airflow limitation (FEV1/FVC <0.70) (16). ACO was defined as coexisting physician-documented diagnoses of asthma and COPD; patients with asthma without a documented COPD diagnosis constituted the comparison group. Published ACO definitions vary, and cigarette smoking is not a mandatory component of all definitions (5). In this retrospective cohort, post-bronchodilator timing, complete exposure histories, and central diagnostic adjudication were not uniformly documented. As shown in Figure 1, 680 patients were screened. We excluded patients younger than 5 years (*n* = 12), those with autoimmune disease or malignancy (*n* = 18), those with missing key clinical or laboratory data (*n* = 55), those with other major respiratory diseases such as interstitial lung disease or active pulmonary tuberculosis (*n* = 13), those in whom COPD could not be confirmed (*n* = 28), those who did not meet Global Initiative for Asthma diagnostic criteria (*n* = 19), and records that failed prespecified data-quality checks (*n* = 30). The final analytic cohort comprised 505 patients, including 190 (37.6%) with ACO and 315 (62.4%) with asthma without COPD.

**Figure 1:**
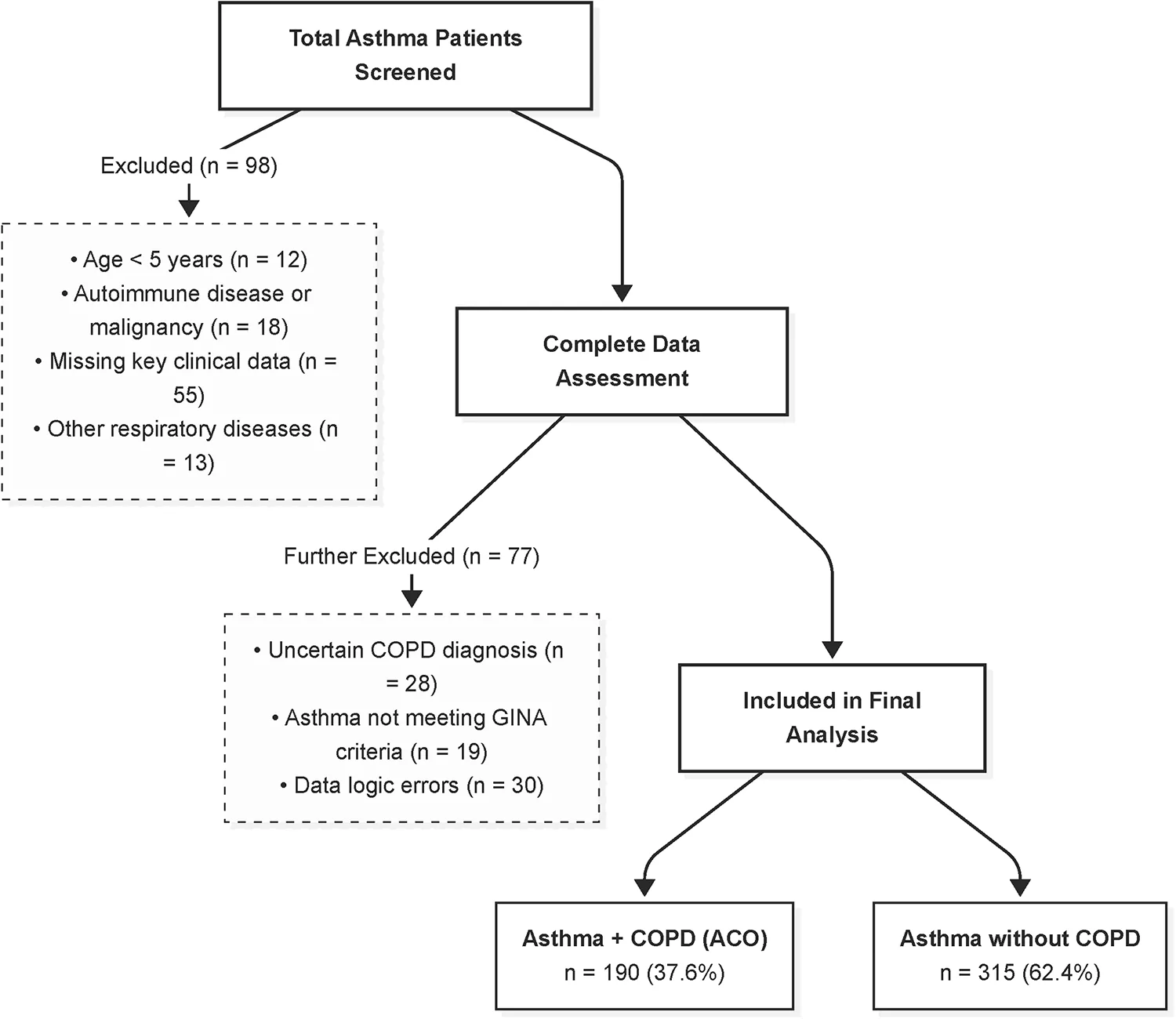
Study flow diagram. Of 680 patients with asthma who were screened, 175 were excluded and 505 were included in the analysis: 190 with asthma–COPD overlap (ACO) and 315 with asthma without COPD. COPD, chronic obstructive pulmonary disease; GINA, global initiative for asthma.

### Data collection and variable definitions

Demographic and clinical variables were extracted from electronic medical records, including age, sex, body mass index (BMI), smoking status, disease duration, asthma type (cough-variant asthma or other), acute severity, allergy history, pulmonary function measures, complete blood count parameters, and recorded respiratory medications. Pulmonary function variables included vital capacity, forced vital capacity, forced expiratory volume in 1 s (FEV1), FEV1/FVC, peak expiratory flow, total lung capacity, and diffusing capacity of the lungs for carbon monoxide. Medication variables included inhaled corticosteroids (ICS), long-acting β2-agonists (LABA), long-acting muscarinic antagonists (LAMA), leukotriene receptor antagonists, theophylline, systemic corticosteroids, and other bronchodilators. NLR was calculated as the absolute neutrophil count divided by the absolute lymphocyte count. dNLR was calculated as the absolute neutrophil count divided by total white blood cell count minus absolute neutrophil count (6, 7). Both markers were analyzed as continuous variables, standardized variables, and quartiles. NLR quartiles were Q1 [0.408, 1.37), Q2 [1.37, 1.90), Q3 [1.90, 2.79), and Q4 [2.79, 20.8]. dNLR quartiles were Q1 [0.217, 0.945), Q2 [0.945, 1.30), Q3 [1.30, 1.86), and Q4 [1.86, 16.6]. The asthma-type variable was a secondary record-based subtype and did not determine eligibility. Acute severity was the mild, moderate, or severe category recorded at the index encounter and was not used as a measure of recent exacerbation history. Smoking was coded as a binary history variable. Pack-year information was recorded when documented in the medical record. A pragmatic blood eosinophilic phenotype was defined as a peripheral blood eosinophil count ≥0.30 × 10^9^/L (≥300 cells/μL); a threshold of ≥0.15 × 10^9^/L was examined in sensitivity analysis, consistent with thresholds used in severe eosinophilic asthma studies (17). Estimated age at asthma onset was calculated as age at the index encounter minus disease duration; one derived value below zero was excluded. Allergy history was included as the comorbidity-related covariate in Model 3 because it represents a spectrum of coexisting allergic diseases that are common in patients with asthma.

### Statistical analysis

Continuous variables were assessed for normality using the Shapiro–Wilk test. Normally distributed variables are reported as mean ± standard deviation, and skewed variables as median (first quartile, third quartile). Differences across quartiles were assessed using one-way analysis of variance or the Kruskal–Wallis test, as appropriate. Categorical variables are reported as number (percentage) and were compared using the Pearson chi-square test or Fisher exact test. Missing data were handled using multivariate imputation by chained equations, and inferential analyses were based on a single imputation. Associations with ACO prevalence were evaluated using logistic regression. Because of the cross-sectional design, odds ratios were interpreted as prevalence odds ratios. Age, estimated age at asthma onset, allergy history, blood eosinophil counts, and blood eosinophilic phenotype frequencies were compared between groups. Fully adjusted logistic models estimated the association between blood eosinophilic phenotype and ACO prevalence and tested phenotype-by-NLR and phenotype-by-dNLR interaction terms. Model 1 was unadjusted. Model 2 adjusted for age, sex, BMI, and smoking. Model 3 further adjusted for acute severity, disease duration, allergy status, and asthma type. Results are reported as odds ratios (ORs) with 95% confidence intervals (CIs). Restricted cubic spline models used four knots at the 5th, 35th, 65th, and 95th percentiles, with the median as the reference. Nonlinearity was evaluated using a likelihood-ratio test. When evidence of nonlinearity was present, two-piecewise logistic regression was used to estimate a data-derived inflection point by maximizing the model log likelihood. A sensitivity model also adjusted for recorded systemic corticosteroid exposure. A stricter spirometric-proxy analysis compared patients with physician-documented COPD, age at least 40 years, and recorded FEV1/FVC below 0.70, consistent with the GOLD fixed-ratio criterion (16), with patients without documented COPD; physician-COPD records not meeting the proxy were excluded. Medication-stratified analyses included multiplicative interaction terms and were considered exploratory. All tests were two-sided, and *P* < 0.05 was considered statistically significant. Analyses were performed using R, version 4.2.1.

## Results

### Baseline characteristics

Baseline characteristics of the 505 participants according to NLR and dNLR quartiles are shown in Tables 1, 2. The median age was 49 years (interquartile range, 37–57 years), 309 participants (61.2%) were women, and the median BMI was 24.4 kg/m^2^. Across NLR quartiles, sex distribution did not differ statistically (*P* = 0.083). Median diffusing capacity differed across NLR quartiles (*P* = 0.025), decreasing from 107% in Q1 to 99% in Q4. Acute severity also differed across NLR quartiles (*P* = 0.022); the proportion classified as severe was 36.5% in Q1 and 18.9% in Q4. Across dNLR quartiles, age (*P* = 0.398), sex (*P* = 0.156), and BMI (*P* = 0.633) did not differ statistically.

**Table 1:** Participant characteristics according to NLR quartiles.

Characteristic	NLR					*P* value
	Overall *N* = 505	Q1 [0.408,1.37) *N* = 126	Q2 [1.37,1.9) *N* = 126	Q3 [1.9,2.79) *N* = 126	Q4 [2.79,20.8] *N* = 127	
Age, years, Median (Q1, Q3)	49 (37, 57)	50 (37, 59)	48 (35, 55)	49 (39, 57)	50 (35, 58)	0.683^a^
Sex, *n* (%)						0.083^b^
Female	309 (61.2%)	84 (66.7%)	81 (64.3%)	78 (61.9%)	66 (52.0%)	
Male	196 (38.8%)	42 (33.3%)	45 (35.7%)	48 (38.1%)	61 (48.0%)	
BMI, kg/m^2^, Median (Q1, Q3)	24.4 (22.0, 27.0)	24.6 (21.9, 26.9)	24.5 (22.1, 27.2)	23.6 (22.1, 27.1)	25.2 (22.1, 27.0)	0.500^a^
Smoking, *n* (%)	136 (26.9%)	35 (27.8%)	32 (25.4%)	42 (33.3%)	27 (21.3%)	0.180^b^
VC%, Mea*n* ± SD	100 ± 17	103 ± 15	101 ± 14	101 ± 19	97 ± 20	0.067^c^
FVC%, Median (Q1, Q3)	101 (90, 113)	103 (92, 116)	101 (89, 111)	101 (89, 112)	98 (88, 111)	0.162^a^
FEV1%, Median (Q1, Q3)	84 (69, 96)	86 (75, 96)	84 (71, 96)	81 (67, 101)	82 (63, 95)	0.254^a^
FEV1/FVC, %, Median (Q1, Q3)	69 (60, 76)	71 (62, 76)	71 (61, 77)	68 (60, 75)	69 (58, 75)	0.412^a^
PEF%, Median (Q1, Q3)	85 (67, 100)	89 (73, 101)	86 (70, 103)	84 (64, 101)	82 (61, 95)	0.068^a^
TLC%, Median (Q1, Q3)	107 (96, 121)	107 (94, 118)	106 (95, 120)	109 (97, 123)	107 (97, 125)	0.736^a^
DLCO%, Median (Q1, Q3)	103 (86, 117)	107 (88, 122)	105 (86, 118)	101 (84, 117)	99 (76, 113)	0.025^a^
IgE, U, Median (Q1, Q3)	118 (54, 461)	139 (54, 515)	126 (53, 359)	136 (54, 515)	97 (44, 434)	0.544^a^
Duration, years, Median (Q1, Q3)	1 (0, 5)	0 (0, 3)	0 (0, 5)	0 (0, 5)	1 (0, 7)	0.195^a^
Asthma Type, *n* (%)						0.127^b^
CVA	108 (21.4%)	32 (25.4%)	25 (19.8%)	32 (25.4%)	19 (15.0%)	
Other	397 (78.6%)	94 (74.6%)	101 (80.2%)	94 (74.6%)	108 (85.0%)	
Acute Severity, *n* (%)						0.022^b^
Mild	137 (27.1%)	38 (30.2%)	30 (23.8%)	32 (25.4%)	37 (29.1%)	
Moderate	213 (42.2%)	42 (33.3%)	51 (40.5%)	54 (42.9%)	66 (52.0%)	
Severe	155 (30.7%)	46 (36.5%)	45 (35.7%)	40 (31.7%)	24 (18.9%)	
Allergy, *n* (%)	273 (54.1%)	65 (51.6%)	72 (57.1%)	68 (54.0%)	68 (53.5%)	0.848^b^
ICS, *n* (%)	329 (65.1%)	81 (64.3%)	78 (61.9%)	77 (61.1%)	93 (73.2%)	0.159^b^
LABA, *n* (%)	394 (78.0%)	100 (79.4%)	95 (75.4%)	98 (77.8%)	101 (79.5%)	0.847^b^
LAMA, *n* (%)	71 (14.1%)	14 (11.1%)	20 (15.9%)	16 (12.7%)	21 (16.5%)	0.555^b^
Leukotriene Receptor Antagonist, *n* (%)	98 (19.4%)	26 (20.6%)	28 (22.2%)	19 (15.1%)	25 (19.7%)	0.517^b^
Theophylline, *n* (%)	68 (13.5%)	18 (14.3%)	16 (12.7%)	18 (14.3%)	16 (12.6%)	0.962^b^
Systemic Corticosteroids, *n* (%)	17 (3.4%)	3 (2.4%)	4 (3.2%)	5 (4.0%)	5 (3.9%)	0.948^d^
Other Bronchodilators, *n* (%)	19 (3.8%)	7 (5.6%)	4 (3.2%)	4 (3.2%)	4 (3.1%)	0.748^d^

a Kruskal–Wallis rank sum test.

b Pearson's Chi-squared test.

c One-way analysis of means.

d Fisher's exact test.

BMI, body mass index; CVA, cough-variant asthma; DLCO, diffusing capacity of the lungs for carbon monoxide; FEV_1_, forced expiratory volume in 1 s; FVC, forced vital capacity; ICS, inhaled corticosteroid; IgE, immunoglobulin E; LABA, long-acting β_2_-agonist; LAMA, long-acting muscarinic antagonist; NLR, neutrophil-to-lymphocyte ratio; PEF, peak expiratory flow; TLC, total lung capacity; VC, vital capacity.

**Table 2:** Participant characteristics according to dNLR quartiles.

Characteristic	dNLR					*P* value
	Overall *N* = 505	Q1 [0.217, 0.945) *N* = 126	Q2 [0.945, 1.3) *N* = 126	Q3 [1.3, 1.86) *N* = 126	Q4 [1.86, 16.6] *N* = 127	
Age, years, Median (Q1, Q3)	49 (37, 57)	50 (39, 58)	48 (34, 56)	49 (41, 58)	49 (35, 58)	0.398^a^
Sex, *n* (%)						0.156^b^
Female	309 (61.2%)	84 (66.7%)	82 (65.1%)	74 (58.7%)	69 (54.3%)	
Male	196 (38.8%)	42 (33.3%)	44 (34.9%)	52 (41.3%)	58 (45.7%)	
BMI, kg/m^2^, Median (Q1, Q3)	24.4 (22.0, 27.0)	24.6 (22.0, 26.8)	23.9 (21.4, 27.0)	24.0 (22.4, 27.3)	25.0 (22.3, 27.0)	0.633^a^
Smoking, *n* (%)	136 (26.9%)	34 (27.0%)	33 (26.2%)	41 (32.5%)	28 (22.0%)	0.309^b^
VC%, Mean ± SD	100 ± 17	102 ± 15	102 ± 14	100 ± 18	98 ± 20	0.147^c^
FVC%, Median (Q1, Q3)	101 (90, 113)	101 (92, 115)	103 (90, 114)	101 (89, 112)	98 (89, 110)	0.323^a^
FEV1%, Median (Q1, Q3)	84 (69, 96)	86 (73, 96)	82 (71, 96)	83 (67, 93)	84 (66, 96)	0.691^a^
FEV1/FVC, %, Median (Q1, Q3)	69 (60, 76)	71 (61, 77)	69 (60, 77)	67 (60, 75)	69 (59, 76)	0.726^a^
PEF%, Median (Q1, Q3)	85 (67, 100)	88 (69, 101)	86 (68, 100)	83 (64, 101)	84 (68, 97)	0.418^a^
TLC%, Median (Q1, Q3)	107 (96, 121)	108 (94, 120)	107 (98, 121)	106 (97, 120)	106 (95, 125)	0.920^a^
DLCO%, Median (Q1, Q3)	103 (86, 117)	105 (82, 119)	106 (91, 121)	100 (82, 113)	102 (79, 117)	0.149^a^
IgE, U, Median (Q1, Q3)	118 (54, 461)	189 (55, 515)	139 (54, 545)	85 (53, 285)	100 (44, 608)	0.191^a^
Duration, years, Median (Q1, Q3)	1 (0, 5)	1 (0, 3)	0 (0, 5)	0 (0, 5)	1 (0, 5)	0.831^a^
Asthma Type, *n* (%)						0.456^b^
CVA	108 (21.4%)	32 (25.4%)	23 (18.3%)	29 (23.0%)	24 (18.9%)	
Other	397 (78.6%)	94 (74.6%)	103 (81.7%)	97 (77.0%)	103 (81.1%)	
Acute Severity, *n* (%)						0.178^b^
Mild	137 (27.1%)	35 (27.8%)	30 (23.8%)	36 (28.6%)	36 (28.3%)	
Moderate	213 (42.2%)	45 (35.7%)	52 (41.3%)	53 (42.1%)	63 (49.6%)	
Severe	155 (30.7%)	46 (36.5%)	44 (34.9%)	37 (29.4%)	28 (22.0%)	
Allergy, *n* (%)	273 (54.1%)	64 (50.8%)	73 (57.9%)	65 (51.6%)	71 (55.9%)	0.618^b^
ICS, *n* (%)	329 (65.1%)	84 (66.7%)	72 (57.1%)	82 (65.1%)	91 (71.7%)	0.109^b^
LABA, *n* (%)	394 (78.0%)	102 (81.0%)	88 (69.8%)	102 (81.0%)	102 (80.3%)	0.087^b^
LAMA, *n* (%)	71 (14.1%)	10 (7.9%)	18 (14.3%)	22 (17.5%)	21 (16.5%)	0.124^b^
Leukotriene Receptor Antagonist, *n* (%)	98 (19.4%)	25 (19.8%)	26 (20.6%)	20 (15.9%)	27 (21.3%)	0.700^b^
Theophylline, *n* (%)	68 (13.5%)	15 (11.9%)	15 (11.9%)	18 (14.3%)	20 (15.7%)	0.761^b^
Systemic Corticosteroids, *n* (%)	17 (3.4%)	3 (2.4%)	5 (4.0%)	6 (4.8%)	3 (2.4%)	0.679^d^
Other Bronchodilators, *n* (%)	19 (3.8%)	7 (5.6%)	6 (4.8%)	1 (0.8%)	5 (3.9%)	0.147^d^

a Kruskal–Wallis rank sum test.

b Pearson's Chi-squared test.

c One-way analysis of means.

d Fisher's exact test.

BMI, body mass index; CVA, cough-variant asthma; DLCO, diffusing capacity of the lungs for carbon monoxide; dNLR, derived neutrophil-to-lymphocyte ratio; FEV_1_, forced expiratory volume in 1 s; FVC, forced vital capacity; ICS, inhaled corticosteroid; IgE, immunoglobulin E; LABA, long-acting β_2_-agonist; LAMA, long-acting muscarinic antagonist; PEF, peak expiratory flow; TLC, total lung capacity; VC, vital capacity.

Using the ≥0.30 × 10^9^/L threshold, 254/505 patients (50.3%) had a blood eosinophilic phenotype, including 80/190 (42.1%) with ACO and 174/315 (55.2%) with asthma without COPD (*P* = 0.004). Median blood eosinophil counts were 0.22 × 10^9^/L (IQR, 0.09–0.54) and 0.35 × 10^9^/L (IQR, 0.11–0.63), respectively (*P* = 0.015). After multivariable adjustment, the blood eosinophilic phenotype was not independently associated with ACO prevalence (OR, 0.67; 95% CI, 0.43–1.05; *P* = 0.084). There was no evidence that blood eosinophilic phenotype modified the associations of NLR or dNLR with ACO prevalence (*P* for interaction = 0.866 and 0.714, respectively) (Supplementary Tables S9–S10).

Patients with ACO were older than those with asthma without COPD: median age was 53.0 years (IQR, 42.0–60.0) vs. 47.0 years (IQR, 35.0–54.5; *P* < 0.001). Median estimated age at asthma onset was 50.0 years (IQR, 38.0–57.0) in ACO and 42.0 years (IQR, 32.0–51.0) in asthma without COPD (*P* < 0.001). Allergy history was present in 36/190 patients with ACO (18.9%) and 237/315 patients with asthma without COPD (75.2%; *P* < 0.001). Smoking history was present in 50/190 patients with ACO (26.3%) and 86/315 patients with asthma without COPD (27.3%; *P* = 0.809). Median TLC was 108.5% predicted (IQR, 97.0–123.9) in ACO and 106.0% predicted (IQR, 95.0–119.0) in asthma without COPD (*P* = 0.171). Median DLCO was 96.0% predicted (IQR, 76.0–111.0) in ACO and 107.0% predicted (IQR, 91.0–121.0) in asthma without COPD (*P* < 0.001). Among patients with available pack-year information, median pack-years were 17.5 (IQR, 9.4–26.3) in ACO and 12.0 (IQR, 5.0–20.0) in asthma without COPD; pack-years were not entered into regression models (Supplementary Tables S6–S7).

Among patients without documented COPD, 126/315 (40.0%) had a recorded FEV1/FVC below 0.70 and 13/315 (4.1%) had both FEV1/FVC below 0.70 and negative reversibility. Among 85 patients with severe asthma without COPD, 37 had FEV1/FVC below 0.70 and 6 had both features. These findings show why fixed airflow limitation alone was not used to establish COPD in this retrospective cohort (Supplementary Table S6).

### Associations of NLR and dNLR with ACO prevalence

The logistic regression results are presented in Table 3. In Model 3, each one-unit increase in NLR was associated with higher ACO prevalence (OR, 1.26; 95% CI, 1.14–1.40; *P* < 0.001), and the standardized NLR showed a similar association (OR, 1.80; 95% CI, 1.38–2.35; *P* < 0.001). Compared with NLR Q1, Q4 was associated with higher ACO prevalence (OR, 4.97; 95% CI, 2.50–9.87; *P* < 0.001), with evidence of a trend across quartiles (*P* for trend < 0.001).

**Table 3:** Associations of NLR and dNLR with ACO prevalence.

Characteristic	Model 1: OR (95% CI), *P* value	Model 2: OR (95% CI), *P* value	Model 3: OR (95% CI), *P* value
NLR			
Continuous	1.15 (1.06, 1.26), <0.001	1.16 (1.06, 1.27), <0.001	1.26 (1.14, 1.40), <0.001
Standardized	1.43 (1.16, 1.77), <0.001	1.46 (1.17, 1.82), <0.001	1.80 (1.38, 2.35), <0.001
Quartiles			
** **Q1 [0.408, 1.37)	Ref.	Ref.	Ref.
** **Q2 [1.37, 1.9)	1.55 (0.91, 2.63), 0.108	1.64 (0.95, 2.83), 0.074	2.55 (1.31, 4.95), 0.006
** **Q3 [1.9, 2.79)	1.55 (0.91, 2.63), 0.108	1.59 (0.93, 2.75), 0.093	2.32 (1.20, 4.47), 0.012
** **Q4 [2.79, 20.8]	2.40 (1.42, 4.05), 0.001	2.53 (1.47, 4.37), <0.001	4.97 (2.50, 9.87), <0.001
*P* for trend	0.002	0.002	<0.001
dNLR			
Continuous	1.28 (1.11, 1.49), 0.001	1.30 (1.11, 1.52), 0.001	1.52 (1.25, 1.85), <0.001
Standardized	1.47 (1.17, 1.85), 0.001	1.49 (1.17, 1.90), 0.001	1.91 (1.41, 2.59), <0.001
Quartiles			
** **Q1 [0.217, 0.945)	Ref.	Ref.	Ref.
** **Q2 [0.945, 1.3)	0.96 (0.56, 1.65), 0.890	1.03 (0.59, 1.78), 0.925	1.23 (0.64, 2.37), 0.533
** **Q3 [1.3, 1.86)	2.04 (1.22, 3.42), 0.007	2.02 (1.19, 3.43), 0.009	3.19 (1.66, 6.15), <0.001
** **Q4 [1.86, 16.6]	1.83 (1.09, 3.06), 0.022	1.92 (1.12, 3.28), 0.017	3.31 (1.71, 6.41), <0.001
*P* for trend	0.002	0.002	<0.001

Model 1 was unadjusted. Model 2 adjusted for age, sex, body mass index, and smoking. Model 3 additionally adjusted for acute severity, disease duration, allergy, and asthma type.

ACO, asthma–COPD overlap; CI, confidence interval; dNLR, derived neutrophil-to-lymphocyte ratio; NLR, neutrophil-to-lymphocyte ratio; OR, odds ratio; Ref., reference group.

In Model 3, each one-unit increase in dNLR was associated with higher ACO prevalence (OR, 1.52; 95% CI, 1.25–1.85; *P* < 0.001). Compared with dNLR Q1, Q4 was associated with higher ACO prevalence (OR, 3.31; 95% CI, 1.71–6.41; *P* < 0.001), with evidence of a trend across quartiles (*P* for trend <0.001). Higher white blood cell count and neutrophil count were also associated with higher ACO prevalence in the fully adjusted models (Supplementary Tables S1 and S2). The continuous lymphocyte-count association in Model 3 included the null (OR, 0.76; 95% CI, 0.57–1.00; *P* = 0.052; Supplementary Table S3). Neutrophil percentage was positively associated with ACO prevalence (Supplementary Table S4), whereas lymphocyte percentage was inversely associated (Supplementary Table S5). Because dNLR is a monotonic transformation of neutrophil percentage [neutrophil proportion/(1−neutrophil proportion)], their quartile groupings and categorical ORs were identical.

In sensitivity analyses, adding recorded systemic corticosteroid exposure to Model 3 produced similar associations for NLR (OR, 1.26; 95% CI, 1.13–1.40; *P* < 0.001) and dNLR (OR, 1.51; 95% CI, 1.24–1.84; *P* < 0.001). The stricter spirometric-proxy analysis included 432 patients and 117 ACO proxy events; the adjusted associations remained positive for NLR (OR, 1.33; 95% CI, 1.18–1.49; *P* < 0.001) and dNLR (OR, 1.67; 95% CI, 1.32–2.12; *P* < 0.001) (Supplementary Table S8).

### Nonlinear associations and inflection-point analyses

Restricted cubic spline analyses are shown in Figures 2,3. For NLR, the adjusted prevalence odds increased rapidly at lower values and continued to increase more gradually at higher values (*P* overall <0.001; *P* for nonlinearity = 0.023). The dNLR curve showed a similar pattern (*P* overall <0.001; *P* for nonlinearity = 0.023).

**Figure 2:**
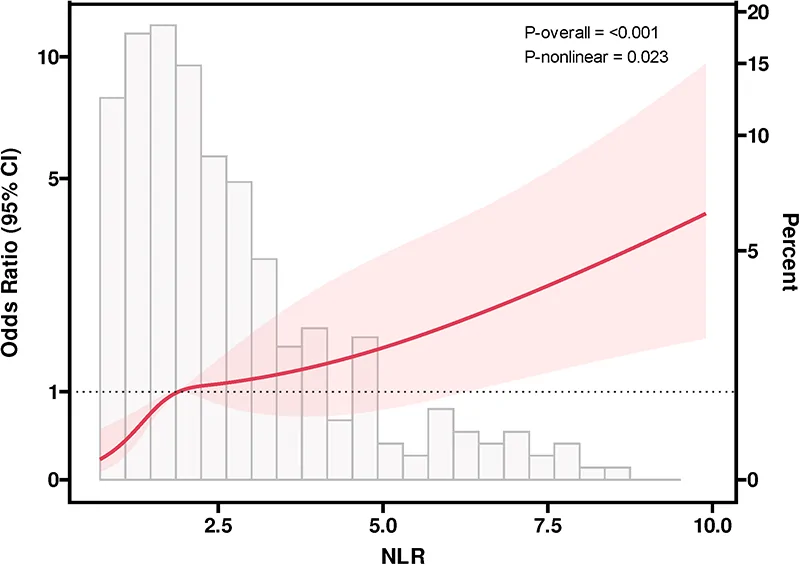
Restricted cubic spline analysis of the association between neutrophil-to-lymphocyte ratio (NLR) and asthma–COPD overlap (ACO) prevalence. The solid red line shows the adjusted odds ratio (OR), the shaded band shows the 95% confidence interval (CI), and the histogram shows the NLR distribution. The model was adjusted for age, sex, body mass index, smoking, asthma type, acute severity, disease duration, and allergy. *P* for nonlinearity = 0.023.

**Figure 3:**
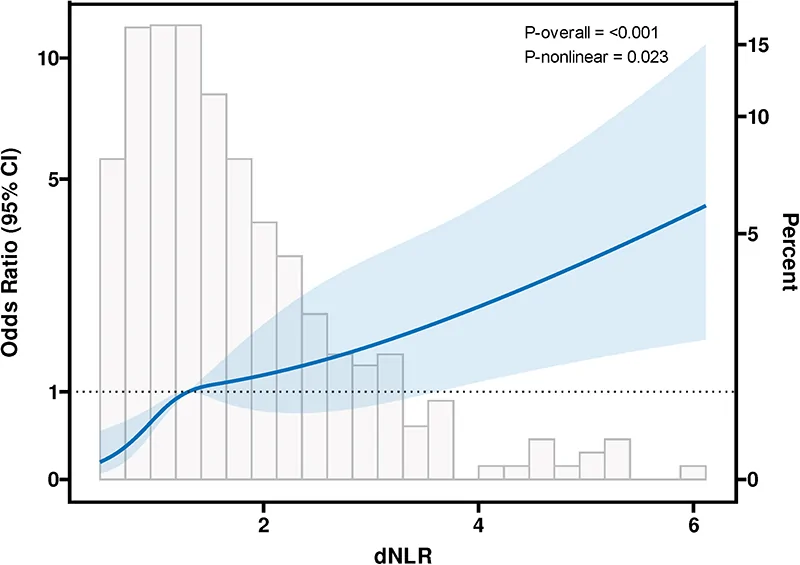
Restricted cubic spline analysis of the association between derived neutrophil-to-lymphocyte ratio (dNLR) and asthma–COPD overlap (ACO) prevalence. The solid blue line shows the adjusted odds ratio (OR), the shaded band shows the 95% confidence interval (CI), and the histogram shows the dNLR distribution. The model was adjusted for age, sex, body mass index, smoking, asthma type, acute severity, disease duration, and allergy. *P* for nonlinearity = 0.023.

Piecewise logistic regression results are shown in Table 4. The estimated NLR inflection point was 2.0. Below 2.0, the OR per one-unit increase was 2.63 (95% CI, 1.44–4.78; *P* = 0.002); at or above 2.0, the corresponding OR was 1.19 (95% CI, 1.07–1.32; *P* = 0.002). The likelihood-ratio test favored the piecewise model (*P* = 0.013). The estimated dNLR inflection point was 1.3. Below 1.3, the OR per one-unit increase was 6.04 (95% CI, 2.17–16.82; *P* < 0.001); at or above 1.3, the OR was 1.33 (95% CI, 1.10–1.61; *P* = 0.003). The likelihood-ratio test also favored the dNLR piecewise model (*P* = 0.006).

**Table 4:** Inflection-point analysis of NLR and dNLR associations with ACO prevalence.

Predictor/Model	Adjusted OR (95% CI)	*P* value
NLR		
Standard Logistic regression	1.26 (1.14, 1.40)	<0.001
Piecewise logistic regression (inflection point = 2.0)		
** **NLR <2	2.63 (1.44, 4.78)	0.002
** **NLR ≥2	1.19 (1.07, 1.32)	0.002
*Log likelihood ratio*		0.013
dNLR		
Standard Logistic regression	1.52 (1.25, 1.85)	<0.001
Piecewise logistic regression (inflection point = 1.3)		
** **dNLR <1.3	6.04 (2.17, 16.82)	<0.001
** **dNLR ≥1.3	1.33 (1.10, 1.61)	0.003
*Log likelihood ratio*		0.006

Models were adjusted for age, sex, body mass index, smoking, asthma type, acute severity, disease duration, and allergy.

ACO, asthma–COPD overlap; CI, confidence interval; dNLR, derived neutrophil-to-lymphocyte ratio; NLR, neutrophil-to-lymphocyte ratio; OR, odds ratio.

### Exploratory medication-stratified analyses

Exploratory medication-stratified results are shown in Figures 4,5. For NLR, interaction tests were significant for ICS (*P* for interaction = 0.028), LAMA (*P* for interaction = 0.032), and ICS plus LABA use (*P* for interaction = 0.026). The NLR association was stronger among ICS users than nonusers and weaker among LAMA users than nonusers. For dNLR, interaction tests were significant for ICS (*P* for interaction = 0.047), LAMA (*P* for interaction = 0.048), and ICS plus LABA use (*P* for interaction = 0.045). Because treatment was not assigned and several strata were small, these findings should be interpreted as exploratory effect-modification signals rather than treatment effects.

**Figure 4:**
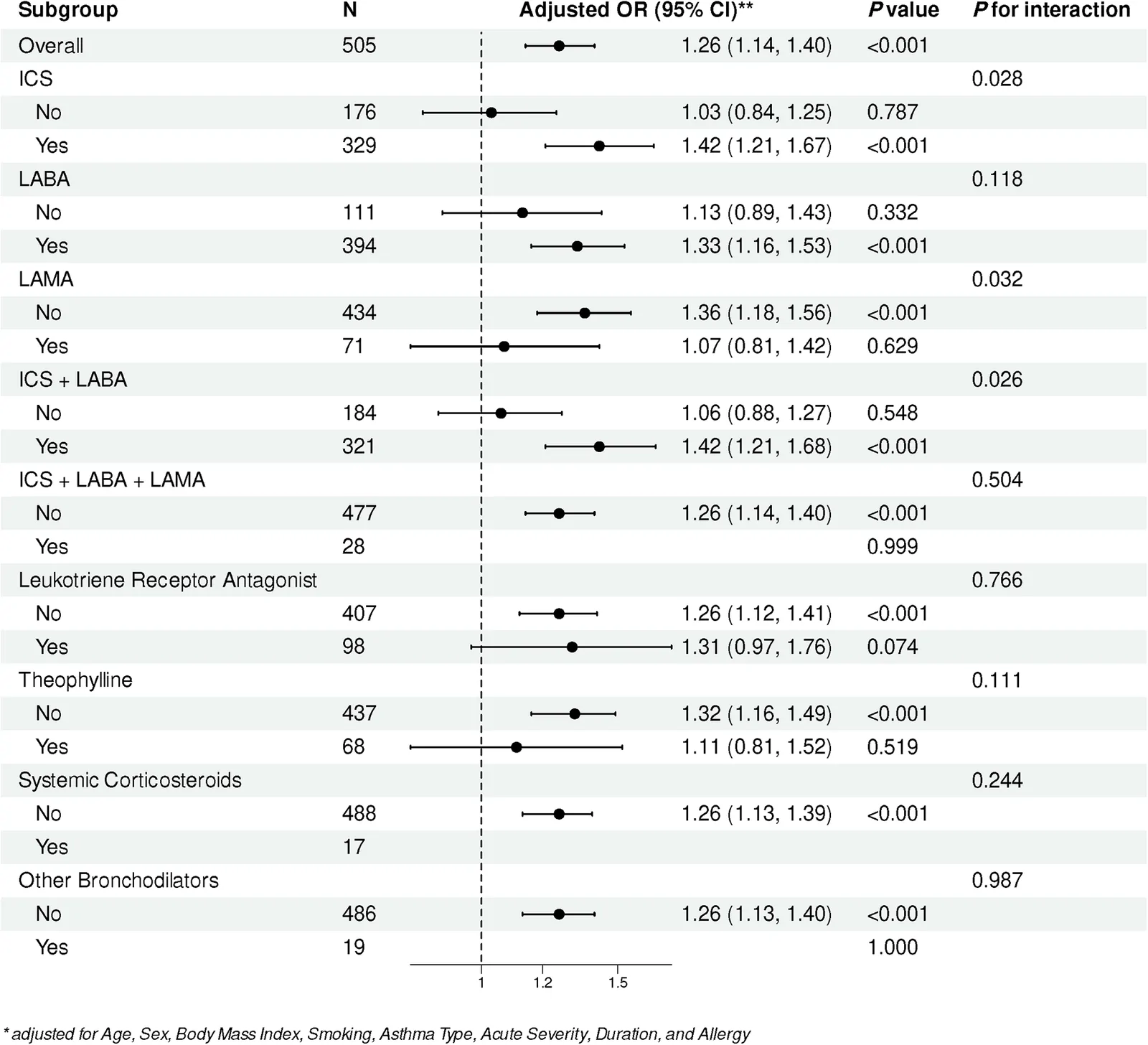
Exploratory medication-stratified analysis of the association between neutrophil-to-lymphocyte ratio (NLR) and asthma–COPD overlap prevalence. Adjusted odds ratios (ORs) and 95% confidence intervals (CIs) are shown for each stratum. Models were adjusted for age, sex, body mass index, smoking, asthma type, acute severity, disease duration, and allergy, except for the stratification variable. Blank estimates indicate nonconvergence in small strata. ICS, inhaled corticosteroid; LABA, long-acting β_2_-agonist; LAMA, long-acting muscarinic antagonist.

**Figure 5:**
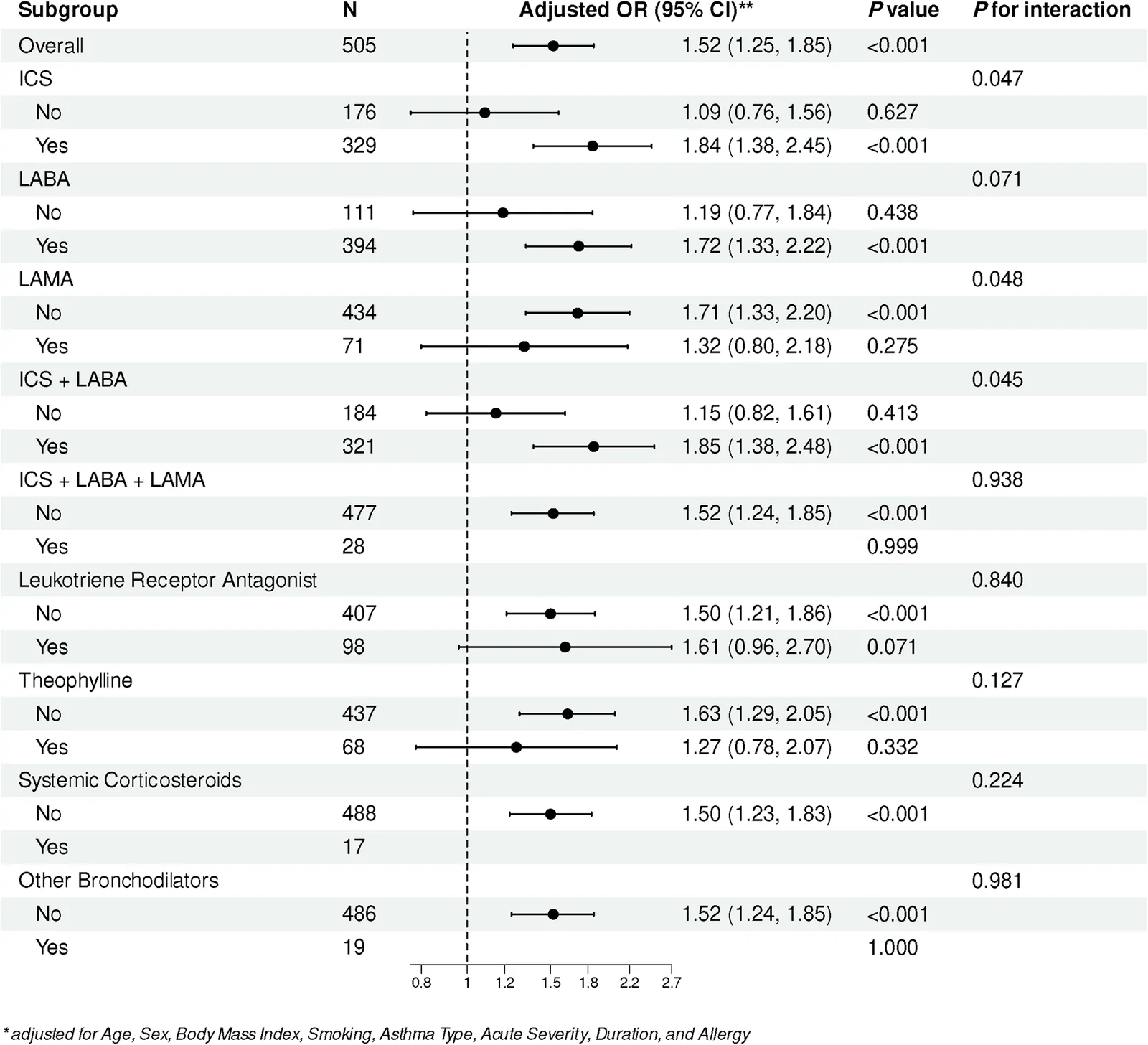
Exploratory medication-stratified analysis of the association between derived neutrophil-to-lymphocyte ratio (dNLR) and asthma–COPD overlap prevalence. Adjusted odds ratios (ORs) and 95% confidence intervals (CIs) are shown for each stratum. Models were adjusted for age, sex, body mass index, smoking, asthma type, acute severity, disease duration, and allergy, except for the stratification variable. Blank estimates indicate nonconvergence in small strata. ICS, inhaled corticosteroid; LABA, long-acting β_2_-agonist; LAMA, long-acting muscarinic antagonist.

## Discussion

In this retrospective cross-sectional cohort of patients with asthma, higher NLR and dNLR were associated with higher ACO prevalence after adjustment for measured demographic and clinical factors. The associations were nonlinear: the estimated slopes were steeper below NLR 2.0 and dNLR 1.3 and remained positive but attenuated above those values. The highest quartiles of both markers were associated with substantially higher prevalence odds than the lowest quartiles. These results describe associations with prevalent ACO; they do not establish incident risk, diagnostic accuracy, or causality.

The lower proportion of severe acute presentations in the highest NLR quartile deserves caution. This pattern indicates that NLR should not be interpreted as a general marker of acute asthma severity in this dataset. Acute severity and chronic airflow limitation represent different clinical dimensions, and the cross-sectional design cannot determine whether medication exposure, timing of blood sampling, recent exacerbation, infection, or other factors contributed to the observed distribution. The finding should therefore be treated as descriptive rather than as evidence of distinct inflammatory phenotypes.

Asthma is commonly associated with type 2 inflammatory pathways, whereas COPD more often includes neutrophilic and macrophage-predominant inflammation (18, 19). Previous work has documented systemic inflammation in older adults with ACO and has linked NLR with outcomes in COPD and asthma (20–23). Our findings extend this literature by examining the shape of the association within an asthma cohort. However, peripheral blood ratios cannot identify airway-cell phenotypes directly, and the present data do not show that a higher NLR reflects airway remodeling or a transition from asthma to ACO.

Other proposed respiratory biomarkers include sputum measures, YKL-40, platelet-based inflammatory indices, and composite systemic inflammation scores (24–30). NLR and dNLR are inexpensive and readily available, but this practical advantage does not establish superiority over other biomarkers because no head-to-head discrimination analysis was performed. The observed nonlinear pattern is broadly consistent with studies reporting nonlinear NLR associations in COPD, mortality, and pediatric asthma populations (31–33). Nevertheless, the inflection points in the present study were estimated from the same dataset used to assess the associations and should not be regarded as validated clinical cutoffs.

Strengths of this study include evaluation of NLR and dNLR in several forms, multivariable adjustment, and explicit assessment of nonlinearity. Several limitations are important. First, the cross-sectional design precludes temporal or causal inference. Longitudinal follow-up was not available, preventing assessment of temporal direction and changes in treatment or disease status. Second, ACO classification relied on retrospective physician documentation. Although the usual GINA- and GOLD-based diagnostic criteria were specified, incomplete clinical documentation and diagnostic misclassification cannot be excluded; post-bronchodilator timing, complete exposure histories, and central diagnostic adjudication were not uniformly documented. Peripheral blood eosinophils permitted a blood eosinophilic classification, but induced-sputum cytology, FeNO, repeated eosinophil counts, and treatment-timed measurements were unavailable; therefore, complete airway inflammatory phenotyping and stable Type 2 classification were not possible. Estimated age at asthma onset was derived from age and disease duration rather than directly recorded; it is therefore sensitive to recall error, and one implausible negative value was excluded. Other comorbidities will be evaluated more systematically in future studies. Third, missing data were handled using multivariate imputation by chained equations, and inferential analyses were based on a single imputation; therefore, between-imputation uncertainty was not incorporated into the confidence intervals. Missingness was substantial for several clinically relevant variables, including acute severity, smoking, TLC, DLCO, and COPD status. Acute severity was incompletely documented and could not be used as a measure of recent exacerbation history. Fourth, this was a single-center study without external validation. Fifth, residual confounding is likely, including confounding by indication in medication-stratified analyses and possible effects of infection, corticosteroid exposure, adherence, other inflammatory conditions, and incomplete exposure histories. Recent exacerbations were not recorded with a defined look-back interval or event count. Only one record explicitly documented respiratory infection, and its timing relative to blood sampling was unavailable, preventing meaningful adjustment. Pack-year information was incomplete, and corticosteroid dose or timing and persistent treatment history were unavailable. Sixth, several interaction strata were small, and multiple exploratory interaction tests increase the possibility of chance findings. Seventh, dNLR is mathematically determined by neutrophil percentage and therefore provides limited independent information beyond that component.

Clinically, NLR and dNLR may serve as low-cost signals that prompt a review of symptoms, exposure history, medication use, and confirmatory lung-function testing. They should not be used alone to diagnose ACO or to select treatment. Although LAMA-containing regimens have established roles in selected patients with asthma and COPD (34–37), the exploratory medication-interaction findings do not demonstrate that NLR identifies patients who will benefit from LAMA or triple therapy. The data-derived inflection points require independent validation, and the medication-interaction findings are hypothesis-generating and are not clinically actionable. Prospective multicenter studies should validate the nonlinear associations, prespecify thresholds, use a clearly documented post-bronchodilator reference standard, and evaluate incremental discrimination, calibration, and clinical utility beyond routine clinical variables.

## Conclusion

Higher NLR and dNLR were independently associated with higher ACO prevalence in this single-center cohort of patients with asthma. The associations were nonlinear, with steeper slopes below NLR 2.0 and dNLR 1.3 and attenuated positive slopes above those values. These blood-derived indices may help identify patients who warrant further assessment, but they are not stand-alone diagnostic tests, and the proposed inflection points require external prospective validation.

## Data Availability

The raw data supporting the conclusions of this article will be made available by the authors, without undue reservation.

## References

[1] Venkatesan P. GOLD COPD report: 2024 update. Lancet Respir Med. (2024) 12:15–6. 10.1016/S2213-2600(23)00461-7 38061380

[2] Venkatesan P. 2025 GINA report for asthma. Lancet Respir Med. (2025) 13:e41–2. 10.1016/S2213-2600(25)00242-5 40582369

[3] Sin DD Miravitlles M Mannino DM Soriano JB Price D Celli BR. What is asthma-COPD overlap syndrome? Towards a consensus definition from a round table discussion. Eur Respir J. (2016) 48:664–73. 10.1183/13993003.00436-2016 27338195

[4] Christenson SA Smith BM Bafadhel M Putcha N. Chronic obstructive pulmonary disease. Lancet. (2022) 399:2227–42. 10.1016/S0140-6736(22)00470-6 35533707

[5] Alshabanat A Zafari Z Albanyan O Dairi M FitzGerald JM. Asthma and COPD overlap syndrome (ACOS): a systematic review and meta analysis. PLoS One. (2015) 10:e0136065. 10.1371/journal.pone.0136065 26336076 PMC4559416

[6] Ellingsen J Janson C Bröms K Lisspers K Ställberg B Högman M. Neutrophil-to-lymphocyte ratio, blood eosinophils and COPD exacerbations: a cohort study. ERJ Open Res. (2021) 7:00471-2021. 10.1183/23120541.00471-2021 34988219 PMC8711083

[7] Gasmi M Torabinasab K Williams-Hooker R Marsigliante S Muscella A. The neutrophil-to-lymphocyte ratio as a marker of immunosenescence and COVID-19 outcomes in the elderly: a narrative review. Physiol Rep. (2026) 14:e70682. 10.14814/phy2.70682 41673927 PMC12894097

[8] Paliogiannis P Fois AG Sotgia S Mangoni AA Zinellu E Pirina P. Neutrophil to lymphocyte ratio and clinical outcomes in COPD: recent evidence and future perspectives. Eur Respir Rev. (2018) 27:170113. 10.1183/16000617.0113-2017 29436405 PMC9488932

[9] Yang AP Liu JP Tao WQ Li HM. The diagnostic and predictive role of NLR, d-NLR and PLR in COVID-19 patients. Int Immunopharmacol. (2020) 84:106504. 10.1016/j.intimp.2020.106504 32304994 PMC7152924

[10] Furutate R Ishii T Motegi T Hattori K Kusunoki Y Gemma A. The neutrophil to lymphocyte ratio is related to disease severity and exacerbation in patients with chronic obstructive pulmonary disease. Intern Med. (2016) 55:223–29. 10.2169/internalmedicine.55.5772 26831014

[11] Xiong W Xu M Zhao Y Wu X Pudasaini B Liu JM. Can we predict the prognosis of COPD with a routine blood test? Int J Chron Obstruct Pulmon Dis. (2017) 12:615–25. 10.2147/COPD.S124041 28243079 PMC5315204

[12] Jin L Guo J Deng K Yao Y. Neutrophil-to-lymphocyte ratio as a biomarker for asthma identification and severity stratification: a systematic review and meta-analysis. Front Med (Lausanne). (2025) 12:1620695. 10.3389/fmed.2025.1620695 40630485 PMC12236102

[13] Zinellu A Paliogiannis P Sotgiu E Mellino S Mangoni AA Zinellu E. Blood cell count derived inflammation indexes in patients with idiopathic pulmonary fibrosis. Lung. (2020) 198:821–27. 10.1007/s00408-020-00386-7 32844257 PMC7502432

[14] Ma H Yang L Liu L Zhou Y Guo X Wu S. Using inflammatory index to distinguish asthma, asthma-COPD overlap and COPD: a retrospective observational study. Front Med (Lausanne). (2022) 9:1045503. 10.3389/fmed.2022.1045503 36465915 PMC9714673

[15] Guo S. Logistic regression analysis of clinical characteristics for differentiation of chronic obstructive pulmonary disease severity. Emerg Med Int. (2023) 2023:5945191. 10.1155/2023/5945191 36816328 PMC9931480

[16] Agustí A Celli BR Criner GJ Halpin D Anzueto A Barnes P. Global initiative for chronic obstructive lung disease 2023 report: GOLD executive summary. Eur Respir J. (2023) 61:2300239. 10.1183/13993003.00239-2023 PMC10066569 36858443

[17] Ortega HG Yancey SW Mayer B Gunsoy NB Keene ON Bleecker ER. Severe eosinophilic asthma treated with mepolizumab stratified by baseline eosinophil thresholds: a secondary analysis of the DREAM and MENSA studies. Lancet Respir Med. (2016) 4:549–56. 10.1016/S2213-2600(16)30031-5 27177493

[18] Barnes PJ. Immunology of asthma and chronic obstructive pulmonary disease. Nat Rev Immunol. (2008) 8:183–92. 10.1038/nri2254 18274560

[19] Locksley RM. Asthma and allergic inflammation. Cell. (2010) 140:777–83. 10.1016/j.cell.2010.03.004 20303868 PMC3134388

[20] Fu JJ McDonald VM Gibson PG Simpson JL. Systemic inflammation in older adults with asthma-COPD overlap syndrome. Allergy Asthma Immunol Res. (2014) 6:316–24. 10.4168/aair.2014.6.4.316 24991455 PMC4077958

[21] Huang WJ Huang GT Zhan QM Chen JL Luo WT Wu LH. The neutrophil to lymphocyte ratio as a novel predictor of asthma and its exacerbation: a systematic review and meta-analysis. Eur Rev Med Pharmacol Sci. (2020) 24:11719–28. 10.26355/eurrev_202011_23819 33275241

[22] Dymicka-Piekarska V Dorf J Milewska A Łukaszyk M Kosidło JW Kamińska J. Neutrophil/lymphocyte ratio (NLR) and lymphocyte/monocyte ratio (LMR)—risk of death inflammatory biomarkers in patients with COVID-19. J Inflamm Res. (2023) 16:2209–22. 10.2147/JIR.S409871 37250103 PMC10224725

[23] Erbay M Ayçiçek O Hocek AB Özdemir KN. The diagnostic value of bronchodilator response in differentiating asthma, COPD, and ACO in fixed airflow obstruction: a retrospective study. BMC Pulm Med. (2025) 25:291. 10.1186/s12890-025-03764-0 40604764 PMC12220476

[24] Ding B DiBonaventura M Karlsson N Ling X. Asthma-chronic obstructive pulmonary disease overlap syndrome in the urban Chinese population: prevalence and disease burden using the 2010, 2012, and 2013 China national health and wellness surveys. Int J Chron Obstruct Pulmon Dis. (2016) 11:1139–50. 10.2147/COPD.S103873 27354777 PMC4907484

[25] Gao J Iwamoto H Koskela J Alenius H Hattori N Kohno N. Characterization of sputum biomarkers for asthma-COPD overlap syndrome. Int J Chron Obstruct Pulmon Dis. (2016) 11:2457–65. 10.2147/COPD.S113484 27757028 PMC5053388

[26] Tong X Wang D Liu S Ma Y Li Z Tian P. The YKL-40 protein is a potential biomarker for COPD: a meta-analysis and systematic review. Int J Chron Obstruct Pulmon Dis. (2018) 13:409–18. 10.2147/COPD.S152655 29430175 PMC5796800

[27] Liu L Zhang X Liu Y Zhang L Zheng J Wang J. Chitinase-like protein YKL-40 correlates with inflammatory phenotypes, anti-asthma responsiveness and future exacerbations. Respir Res. (2019) 20:95. 10.1186/s12931-019-1051-9 31113430 PMC6530174

[28] Fois AG Paliogiannis P Scano V Cau S Babudieri S Perra R. The systemic inflammation index on admission predicts in-hospital mortality in COVID-19 patients. Molecules. (2020) 25:5725. 10.3390/molecules25235725 33291581 PMC7731255

[29] Ghobadi H Mohammadshahi J Javaheri N Fouladi N Mirzazadeh Y Aslani MR. Role of leukocytes and systemic inflammation indexes (NLR, PLR, MLP, dNLR, NLPR, AISI, SIR-I, and SII) on admission predicts in-hospital mortality in non-elderly and elderly COVID-19 patients. Front Med (Lausanne). (2022) 9:916453. 10.3389/fmed.2022.916453 36059829 PMC9434555

[30] Yang L Zeng T Li N Jiang D. Association between platelet-to-neutrophil ratio and asthma–COPD overlap: a cross-sectional study in China. Front Med (Lausanne). (2026) 13:2026. 10.3389/fmed.2026.1729278 PMC13039030 41930131

[31] Chen Z Li W Tang Y Zhou P He Q Deng Z. The neutrophil-lymphocyte ratio predicts all-cause and cardiovascular mortality among United States adults with COPD: results from NHANES 1999–2018. Front Med (Lausanne). (2024) 11:1443749. 10.3389/fmed.2024.1443749 39386755 PMC11461198

[32] Cheng C Zhang L. Nonlinear association between neutrophil-to-lymphocyte ratio and asthma in children and adolescents in the United States: a cross-sectional study. Clin Exp Pediatr. (2025) 68:489–96. 10.3345/cep.2024.01844 40083098 PMC12235342

[33] Li C Ao T Huang Y Ding L Zhen P Hu M. Association between neutrophil to lymphocyte ratio/serum albumin ratio and chronic obstructive pulmonary disease: results from NHANES 1999–2018. Int J Chron Obstruct Pulmon Dis. (2025) 20:3951–64. 10.2147/COPD.S559567 41377482 PMC12687652

[34] Gosens R Zaagsma J Meurs H Halayko AJ. Muscarinic receptor signaling in the pathophysiology of asthma and COPD. Respir Res. (2006) 7:73. 10.1186/1465-9921-7-73 16684353 PMC1479816

[35] Kew KM Dahri K. Long-acting muscarinic antagonists (LAMA) added to combination long-acting beta2-agonists and inhaled corticosteroids (LABA/ICS) versus LABA/ICS for adults with asthma. Cochrane Database Syst Rev. (2016) 2016:Cd011721. 10.1002/14651858.CD011721 26798035 PMC9440477

[36] Rayner DG Bakaa L Hoyte F Perry TT Rivera-Spoljaric K Sumino K. Triple therapy vs dual inhaler therapy for moderate-to-severe asthma: an updated systematic review and meta-analysis. Ann Allergy Asthma Immunol. (2025) 135:278–86.e11. 10.1016/j.anai.2025.06.011 40516649

[37] van Smoorenburg LH Karp T Ditz B Van Geffen WH Guryev V Almusa A. Anti-inflammatory effects of tiotropium in COPD: a randomised double-blind trial. ERJ Open Res. (2025) 11:00735-2024. 10.1183/23120541.00735-2024 40166051 PMC11955910

