# Supplementary material for: Nonlinear associations of the neutrophil-to-lymphocyte ratio and derived neutrophil-to-lymphocyte ratio with asthma–COPD overlap prevalence: a cross-sectional study
Source: Front Med (Lausanne). 2026 Sep 29;13:1950041. doi: 10.3389/fmed.2026.1950041 (PMC13658142; doi:10.3389/fmed.2026.1950041)
Supplement: Supplementary file 1 [file Supplementaryfile1.docx]

Table S1: Association between White Blood Cells and ACO Prevalence

| Characteristic | Model 1: OR (95% CI), *P*-value | Model 2: OR (95% CI), *P*-value | Model 3: OR (95% CI), *P*-value |
| --- | --- | --- | --- |
| White Blood Cells |  |  |  |
| Continuous | 1.08 (1.00, 1.17), 0.056 | 1.10 (1.01, 1.19), 0.028 | 1.24 (1.12, 1.37), <0.001 |
| Standardized | 1.19 (1.00, 1.42), 0.056 | 1.23 (1.02, 1.49), 0.028 | 1.61 (1.28, 2.03), <0.001 |
| Quartiles |  |  |  |
| - Q1 [3.25, 5.77) | Ref. | Ref. | Ref. |
| - Q2 [5.77, 6.99) | 1.14 (0.68, 1.91), 0.612 | 1.23 (0.72, 2.09), 0.442 | 1.62 (0.86, 3.06), 0.138 |
| - Q3 [6.99, 8.45) | 1.16 (0.69, 1.95), 0.571 | 1.17 (0.69, 2.00), 0.556 | 2.32 (1.20, 4.48), 0.012 |
| - Q4 [8.45, 18] | 1.36 (0.82, 2.28), 0.236 | 1.47 (0.86, 2.51), 0.155 | 3.05 (1.56, 5.96), 0.001 |
| *P for trend* | 0.252 | 0.196 | <0.001 |

Notes: Model 1: unadjusted. Model 2: adjusted for Age, Sex, BMI, and Smoking. Model 3: adjusted for Age, Sex, BMI, Smoking, Asthma Type, Acute Severity, Duration, and Allergy.

Abbreviations: ACO = Asthma-COPD Overlap; BMI = Body Mass Index; CI = Confidence Interval; OR = Odds Ratio; WBC = White Blood Cells.

Table S2: Association between Neutrophil count and ACO Prevalence

| Characteristic | Model 1: OR (95% CI), *P*-value | Model 2: OR (95% CI), *P*-value | Model 3: OR (95% CI), *P*-value |
| --- | --- | --- | --- |
| Neutrophil |  |  |  |
| Continuous | 1.17 (1.07, 1.28), <0.001 | 1.19 (1.08, 1.31), <0.001 | 1.39 (1.23, 1.57), <0.001 |
| Standardized | 1.37 (1.14, 1.65), <0.001 | 1.42 (1.17, 1.73), <0.001 | 1.95 (1.52, 2.50), <0.001 |
| Quartiles |  |  |  |
| - Q1 [1.1, 3.04) | Ref. | Ref. | Ref. |
| - Q2 [3.04, 3.96) | 2.22 (1.30, 3.81), 0.004 | 2.44 (1.40, 4.24), 0.002 | 3.55 (1.81, 6.96), <0.001 |
| - Q3 [3.96, 4.99) | 1.94 (1.12, 3.33), 0.017 | 2.07 (1.18, 3.63), 0.011 | 4.16 (2.06, 8.39), <0.001 |
| - Q4 [4.99, 17] | 2.50 (1.47, 4.27), <0.001 | 2.73 (1.57, 4.76), <0.001 | 6.75 (3.31, 13.75), <0.001 |
| *P for trend* | 0.003 | 0.002 | <0.001 |

Notes: Model 1: unadjusted. Model 2: adjusted for Age, Sex, BMI, and Smoking. Model 3: adjusted for Age, Sex, BMI, Smoking, Asthma Type, Acute Severity, Duration, and Allergy.

Abbreviations: ACO = Asthma-COPD Overlap; BMI = Body Mass Index; CI = Confidence Interval; OR = Odds Ratio.

Table S3: Association between Lymphocyte count and ACO Prevalence

| Characteristic | Model 1: OR (95% CI), *P*-value | Model 2: OR (95% CI), *P*-value | Model 3: OR (95% CI), *P*-value |
| --- | --- | --- | --- |
| Lymphocyte |  |  |  |
| Continuous | 0.76 (0.60, 0.96), 0.023 | 0.78 (0.61, 0.99), 0.040 | 0.76 (0.57, 1.00), 0.052 |
| Standardized | 0.80 (0.66, 0.97), 0.023 | 0.81 (0.67, 0.99), 0.040 | 0.80 (0.63, 1.00), 0.052 |
| Quartiles |  |  |  |
| - Q1 | Ref. | Ref. | Ref. |
| - Q2 | 0.65 (0.39, 1.09), 0.105 | 0.65 (0.38, 1.10), 0.109 | 0.47 (0.25, 0.90), 0.022 |
| - Q3 | 0.78 (0.47, 1.28), 0.322 | 0.80 (0.48, 1.33), 0.391 | 0.72 (0.38, 1.33), 0.291 |
| - Q4 | 0.61 (0.37, 1.03), 0.063 | 0.66 (0.39, 1.12), 0.123 | 0.66 (0.34, 1.25), 0.201 |
| *P for trend* | 0.120 | 0.222 | 0.416 |

Notes: Model 1: unadjusted. Model 2: adjusted for Age, Sex, BMI, and Smoking. Model 3: adjusted for Age, Sex, BMI, Smoking, Asthma Type, Acute Severity, Duration, and Allergy.

Abbreviations: ACO = Asthma-COPD Overlap; BMI = Body Mass Index; CI = Confidence Interval; OR = Odds Ratio.

Table S4: Association between Neutrophil % and ACO Prevalence

| Characteristic | Model 1: OR (95% CI), *P*-value | Model 2: OR (95% CI), *P*-value | Model 3: OR (95% CI), *P*-value |
| --- | --- | --- | --- |
| Neutrophil % |  |  |  |
| Continuous | 20.43 (4.59, 90.82), <0.001 | 24.15 (5.08, 114.78), <0.001 | 195.47 (26.77, 1427.16), <0.001 |
| Standardized | 1.46 (1.21, 1.76), <0.001 | 1.49 (1.23, 1.82), <0.001 | 1.94 (1.51, 2.49), <0.001 |
| Quartiles |  |  |  |
| - Q1 | Ref. | Ref. | Ref. |
| - Q2 | 0.96 (0.56, 1.65), 0.890 | 1.03 (0.59, 1.78), 0.925 | 1.23 (0.64, 2.37), 0.533 |
| - Q3 | 2.04 (1.22, 3.42), 0.007 | 2.02 (1.19, 3.43), 0.009 | 3.19 (1.66, 6.15), <0.001 |
| - Q4 | 1.83 (1.09, 3.06), 0.022 | 1.92 (1.12, 3.28), 0.017 | 3.31 (1.71, 6.41), <0.001 |
| *P for trend* | 0.002 | 0.002 | <0.001 |

Notes: Model 1: unadjusted. Model 2: adjusted for Age, Sex, BMI, and Smoking. Model 3: adjusted for Age, Sex, BMI, Smoking, Asthma Type, Acute Severity, Duration, and Allergy.

Abbreviations: ACO = Asthma-COPD Overlap; BMI = Body Mass Index; CI = Confidence Interval; OR = Odds Ratio.

Table S5: Association between Lymphocyte % and ACO Prevalence

| Characteristic | Model 1: OR (95% CI), *P*-value | Model 2: OR (95% CI), *P*-value | Model 3: OR (95% CI), *P*-value |
| --- | --- | --- | --- |
| Lymphocyte % |  |  |  |
| Continuous | 0.04 (0.01, 0.25), <0.001 | 0.04 (0.01, 0.24), <0.001 | 0.00 (0.00, 0.04), <0.001 |
| Standardized | 0.72 (0.60, 0.87), <0.001 | 0.71 (0.58, 0.86), <0.001 | 0.55 (0.43, 0.71), <0.001 |
| Quartiles |  |  |  |
| - Q1 | Ref. | Ref. | Ref. |
| - Q2 | 0.75 (0.45, 1.23), 0.252 | 0.76 (0.45, 1.27), 0.288 | 0.55 (0.29, 1.05), 0.069 |
| - Q3 | 0.67 (0.41, 1.12), 0.125 | 0.67 (0.40, 1.13), 0.137 | 0.57 (0.29, 1.09), 0.086 |
| - Q4 | 0.48 (0.29, 0.81), 0.006 | 0.48 (0.28, 0.83), 0.008 | 0.24 (0.12, 0.48), <0.001 |
| *P for trend* | 0.006 | 0.008 | <0.001 |

Notes: Model 1: unadjusted. Model 2: adjusted for Age, Sex, BMI, and Smoking. Model 3: adjusted for Age, Sex, BMI, Smoking, Asthma Type, Acute Severity, Duration, and Allergy.

Abbreviations: ACO = Asthma-COPD Overlap; BMI = Body Mass Index; CI = Confidence Interval; OR = Odds Ratio.

**Table S6. Additional age, allergy history, smoking, lung-function, and airflow-limitation comparisons**

| **Characteristic** | **ACO (n = 190)** | **Asthma without COPD (n = 315)** | **P value** |
| --- | --- | --- | --- |
| Age, years, median (IQR) | 53.0 (42.0-60.0) | 47.0 (35.0-54.5) | <0.001 |
| Estimated age at asthma onset, years, median (IQR) | 50.0 (38.0-57.0) | 42.0 (32.0-51.0) | <0.001 |
| Allergy history, n/N (%) | 36/190 (18.9%) | 237/315 (75.2%) | <0.001 |
| Smoking history, n/N (%) | 50/190 (26.3%) | 86/315 (27.3%) | 0.809 |
| TLC, % predicted, median (IQR) | 108.5 (97.0-123.9) | 106.0 (95.0-119.0) | 0.171 |
| DLCO, % predicted, median (IQR) | 96.0 (76.0-111.0) | 107.0 (91.0-121.0) | <0.001 |
| FEV1/FVC < 0.70, n/N (%) | 144/190 (75.8%) | 126/315 (40.0%) | <0.001 |
| Negative reversibility, n/N (%) | 27/190 (14.2%) | 65/315 (20.6%) | 0.070 |
| FEV1/FVC < 0.70 and negative reversibility, n/N (%) | 16/190 (8.4%) | 13/315 (4.1%) | 0.045 |

Continuous variables are median (IQR); categorical variables are n/N (%).

P values are from Mann-Whitney U tests for continuous variables and Pearson chi-square tests for categorical variables.

Estimated age at asthma onset was calculated as age at the index encounter minus disease duration. One derived value below zero was excluded; the available n was 189 in ACO and 315 in asthma without COPD.

Allergy history represents a spectrum of coexisting allergic diseases that are common in patients with asthma.

Other comorbidities will be evaluated more systematically in future studies.

The FEV1/FVC threshold of 0.70 follows the GOLD fixed-ratio criterion (reference 16 in the main manuscript).

Abbreviations: ACO, asthma-COPD overlap; DLCO, diffusing capacity of the lungs for carbon monoxide; FEV1, forced expiratory volume in 1 second; FVC, forced vital capacity; IQR, interquartile range; TLC, total lung capacity.

**Table S7. Descriptive summary of available pack-year information**

| **Group** | **Median (IQR)** | **Range** |
| --- | --- | --- |
| ACO | 17.5 (9.4-26.3) | 1.5-30.0 |
| Asthma without COPD | 12.0 (5.0-20.0) | 2.5-40.0 |

Available pack-year values were summarized descriptively and were not entered into regression models.

Abbreviations: ACO, asthma-COPD overlap; IQR, interquartile range.

**Table S8. Additional multivariable sensitivity analyses**

| **Analysis** | **Exposure** | **n** | **Events** | **Adjusted OR (95% CI)** | **P value** |
| --- | --- | --- | --- | --- | --- |
| Primary Model 3 | NLR | 505 | 190 | 1.26 (1.14-1.40) | <0.001 |
| Model 3 plus systemic corticosteroid exposure | NLR | 505 | 190 | 1.26 (1.13-1.40) | <0.001 |
| Primary Model 3 | dNLR | 505 | 190 | 1.52 (1.25-1.85) | <0.001 |
| Model 3 plus systemic corticosteroid exposure | dNLR | 505 | 190 | 1.51 (1.24-1.84) | <0.001 |
| Stricter spirometric-proxy outcome | NLR | 432 | 117 | 1.33 (1.18-1.49) | <0.001 |
| Stricter spirometric-proxy outcome | dNLR | 432 | 117 | 1.67 (1.32-2.12) | <0.001 |

Primary Model 3 adjusted for age, sex, body mass index, smoking, asthma type, acute severity, disease duration, and allergy.

The systemic-corticosteroid model also adjusted for recorded systemic corticosteroid exposure.

The stricter proxy required physician-documented COPD, age at least 40 years, and recorded FEV1/FVC below 0.70, consistent with the GOLD fixed-ratio criterion (reference 16 in the main manuscript); physician-COPD records not meeting the proxy were excluded.

Abbreviations: ACO, asthma-COPD overlap; CI, confidence interval; NLR, neutrophil-to-lymphocyte ratio; dNLR, derived neutrophil-to-lymphocyte ratio; OR, odds ratio.

**Table S9. Peripheral-blood eosinophil profile by study group**

| **Characteristic** | **ACO (n = 190)** | **Asthma without COPD (n = 315)** | **P value** |
| --- | --- | --- | --- |
| Blood eosinophil count, ×10^9/L, median (IQR) | 0.22 (0.09-0.54) | 0.35 (0.11-0.63) | 0.015 |
| Blood eosinophils, %, median (IQR) | 3.5 (1.3-6.9) | 5.0 (1.6-8.8) | 0.003 |
| Blood eosinophilic phenotype (EOS ≥0.30 ×10^9/L), n/N (%) | 80/190 (42.1%) | 174/315 (55.2%) | 0.004 |
| Sensitivity threshold (EOS ≥0.15 ×10^9/L), n/N (%) | 125/190 (65.8%) | 225/315 (71.4%) | 0.183 |

Continuous variables are median (IQR); categorical variables are n/N (%).

The primary blood eosinophilic phenotype was defined as EOS ≥0.30 ×10^9/L (≥300 cells/μL); EOS ≥0.15 ×10^9/L was examined as a sensitivity threshold, consistent with thresholds used in severe eosinophilic asthma studies (reference 17 in the main manuscript).

This peripheral-blood classification does not represent induced-sputum or FeNO-based airway inflammatory phenotyping.

Abbreviations: ACO, asthma-COPD overlap; EOS, absolute blood eosinophil count; FeNO, fractional exhaled nitric oxide; IQR, interquartile range.

**Table S10. Adjusted blood eosinophilic phenotype and phenotype-stratified NLR/dNLR analyses**

| **Analysis** | **Population/stratum** | **n/events** | **Adjusted OR (95% CI)** | **P value** | **P for interaction** |
| --- | --- | --- | --- | --- | --- |
| Blood eosinophilic phenotype (EOS ≥0.30 ×10^9/L) | All participants | 505/190 | 0.67 (0.43-1.05) | 0.084 |  |
| Sensitivity threshold (EOS ≥0.15 ×10^9/L) | All participants | 505/190 | 0.66 (0.40-1.07) | 0.092 |  |
| NLR; EOS ≥0.30 ×10^9/L | Non-blood-eosinophilic | 251/110 | 1.27 (1.12-1.44) | <0.001 | 0.866 |
| NLR; EOS ≥0.30 ×10^9/L | Blood eosinophilic | 254/80 | 1.21 (0.98-1.51) | 0.081 | 0.866 |
| dNLR; EOS ≥0.30 ×10^9/L | Non-blood-eosinophilic | 251/110 | 1.49 (1.19-1.86) | <0.001 | 0.714 |
| dNLR; EOS ≥0.30 ×10^9/L | Blood eosinophilic | 254/80 | 1.55 (0.95-2.54) | 0.081 | 0.714 |

All models adjusted for age, sex, body mass index, smoking, asthma type, acute severity, disease duration, and allergy.

Interaction P values test whether the NLR or dNLR association differs between the blood eosinophilic and non-blood-eosinophilic strata.

Abbreviations: ACO, asthma-COPD overlap; CI, confidence interval; dNLR, derived neutrophil-to-lymphocyte ratio; EOS, absolute blood eosinophil count; NLR, neutrophil-to-lymphocyte ratio; OR, odds ratio.
